# Perspectives Emerged from Students and Supervisory Staff Interaction in Drug Use Prevention: A Q Methodology Investigation

**DOI:** 10.3390/ijerph17155621

**Published:** 2020-08-04

**Authors:** Chiu-Mieh Huang, Jung-Yu Liao, Hsiao-Pei Hsu, Cheng-Yu Lin, Jong-Long Guo

**Affiliations:** 1 Institute of Clinical Nursing, School of Nursing, National Yang-Ming University, Taipei 112, Taiwan; cmhuang@ym.edu.tw; 2 Institute of Population Health Sciences, National Health Research Institutes, Miaoli 350, Taiwan; jyliao@nhri.edu.tw; 3 Department of Nursing, School of Nursing, National Yang-Ming University, Taipei 112, Taiwan; sandylucy616@yahoo.com.tw; 4 Department of Radio, Television & Film, Shih Hsin University, Taipei 116, Taiwan; cyou.lin@msa.hinet.net; 5 Department of Health Promotion and Health Education, College of Education, National Taiwan Normal University, Taipei 106, Taiwan

**Keywords:** high school students, drug use prevention, school-based program, Q methodology

## Abstract

This study aims to identify and describe the patterns of shared perspectives of students and supervisory staff associated with their interaction in drug use prevention. We applied the Q methodology to cluster participants into groups according to the similarities of their Q sorts. A total of 31 pairs of students and their supervisory staff participated in the study to rank the designed Q statements. The Q factor analysis for supervisory staff revealed a five-factor solution that accounted for 58% of the total variance. Another five-factor solution for the students explained 49% of the total variance. One similarity between the groups was the need to enhance the involvement of significant others to help the students quit drugs. A major identified difference between the groups was that whereas the students highlighted the importance of health consequences of drug use in helping them stop use, the supervisory staff did not. The elucidation of similarities and differences between supervisory staff and students could offer more insightful strategies of preventing the drug use.

## 1. Introduction

Many drugs contain psychoactive substances and toxic chemicals that are detrimental to human beings, particularly adolescents. The use of these drugs by adolescents is associated with poor neurocognitive performance [1] and brain function [2]. Higher levels of drug use during adolescence predict drug abuse and dependence in adulthood [3]. Recently, campus drug use prevention encountered a critical challenge. The use of novel psychoactive substances (NPS) or so-called “legal highs” has grown rapidly [4]. NPS may be labelled “not for human consumption” to avoid regulation. Many NPS are created by minor alterations in the chemical structure of traditional drugs [5]. Such alterations make the NPS users unaware of what they have consumed. As a result, these effects may lead to serious adverse health consequences. Thus, the importance of finding countermeasures for drug prevention among adolescents is urgent.

The adolescents who exhibit more knowledge about and positive attitudes toward drug use prevention are more likely to avoid drug use [6,7]. Therefore, providing tailored skills and responsive strategies to help adolescents handle possible situations of drug use can effectively decrease the risks of drug use [8,9]. Since adolescents spend much of their time at school, drug use intervention programs are primarily offered in schools to reach students with drug use problems. The results of a meta-analysis examining school-based drug use prevention programs indicated promising effects of these programs on adolescents with substance abuse problems [10].

However, school-based programs targeting students should include strategies to keep students from dropout, which is an essential determinant for the effectiveness of a school-based program. Student dropout from schools often co-occurs with substance use [11]. Empirical studies have recognized that positive youth-adult relationships are beneficial for youth development [12]. Many studies support the association between young people’s positive relationships with adults and improved psychological and behavioral outcomes [13]. Caring adult relationships may be helpful in assisting adolescents to resist the temptation of drug use and in building school bonding, thus preventing students from school dropout. A meta-analytic review of adult mentoring effects on youth delinquency risk found that mentoring interaction had modest positive effects on drug use prevention [14]. A study of examining the effects of youth-adult relationships revealed that program staff with a specific purpose would be even more influential than school teachers [15].

While recognizing the students with drug use problems, building supportive relationships between students and school staff would be a possible solution for preventing students from using drugs again. Perspectives that emerged from students and their supervisory staff would enhance the understanding of how youth-adult relationships can be improved. Regarding youth-adult relationships in drug use prevention for students with drug use problems, both students’ and adults’ perceptions of the interaction process are crucial to understand how positive influences can be effectively delivered. The Q method was successfully used to investigate diverse subjective perceptions in multiple health issues [16,17,18,19,20,21,22]. Instead of evaluating each item separately on a Likert-type scale, individuals were expected to prioritize all items in the Q sort procedure, which requires the agreement degree of all items to be simultaneously evaluated and weighed based on their viewpoints [16]. The Q sort procedure employed the holistic approach and offered an alternative to understand youth-adult relationships in drug use prevention. Hence, the study aimed to identify and describe the groups of shared perspectives associated with supervisory staff and students’ experiences in preventing drug use by applying the Q method.

## 2. Methods

### 2.1. Recruitment

In Taiwan, once a student’s drug use is verified by a urine test, a school staff member is assigned to the student and is scheduled to meet with the student at least once per one or two weeks. During the supervisory period, the staff member has a responsibility to assist the student in remaining drug free for at least 12 weeks. In the current study, we defined the staff member as supervisory staff.

The study adopted the purposive sampling approach by deliberately choosing a particular group of participants due to the qualities these participants possess. It is a nonprobability sampling technique where the participants are gathered in a process that does not give all the participants equal chances of being included [23]. The inclusion criteria for supervisory staff were those who (I) were assisting students with drug use problems during the study period, (II) had completed a 5-day counseling training workshop, (III) were official school personnel during the study period, and (IV) were willing to complete the study and had signed an informed consent form. The inclusion criteria for students were those who (I) were identified as having drug use problems by urine test, (II) were enrolled in a supervisory program to prevent drug use at the time of the study, (III) had no existing cognitive impairment, and (IV) were willing to participate in the study and had signed an informed consent form.

The study recruited supervisory staff from a 5-day counseling training workshop hosted by the Ministry of Education. The workshop aimed to enhance supervisory staff knowledge of the skills required to assist students with drug use problems at vocational and senior high schools. All the attendees (*n* = 100) were invited and informed that their client students would also be invited to participate in the study. Those attendees who were willing to participate (*n* = 38) were provided an information letter detailing the study’s purpose and data collection procedure. The supervisory staff whose client students were not available, since the students had dropped out, were on sick leave, or had declined to participate in the study, were excluded. Subsequently, 31 supervisory staff–student pairs agreed to participate in the study. The participant rate was 81.58% (31/38).

Through supervisory staff, we could have an opportunity to approach these potential student participants. However, we recruited students directly because of the importance of students’ availability to participate, and willingness to communicate supervisory experiences. Students were contacted by one member of the research team, who explained the study in detail and obtained informed consent from the students. Students were free to reject the invitation and decline to participate in the study. The supervisory staff and students were from six vocational and senior high schools: four in New Taipei City, one in Taichung City, and one in Changhua County. All the participants provided written consent.

### 2.2. Research Instruments

#### 2.2.1. Background Information

A structured questionnaire was used to collect participants’ background information. For supervisory staff, data such as their gender, age, duration of services, and duration of employment, were collected. Similarly, data such as gender, age, and status of living with parent were collected from students. Any experience of drug use among students were identified through a urine test. The drugs considered by the study were ketamine, amphetamines, and ecstasy.

#### 2.2.2. Q Methodology

In general, Q methods comprise two techniques: Q sorting procedures and by-person factor analyses [24]. These are also known as Q factor analyses [25]. Designated Q statements were developed to address the area of interest and enable Q sorting usage. Through Q sorting, participants’ perspectives associated with a specific area of interest were investigated by simultaneously ranking all Q statements on a Q sort grid [17]. Rather than evaluating each statement separately on a Likert-type scale, the participants were expected to prioritize the statements in order according to the Q sorting procedure. They weighed the degree of agreement of each statement in relation to other statements. The results of the Q sorts revealed new insights that might not have been elicited using the traditional Likert-type scaling survey [16].

Factor analysis is a technique of data reduction. Application of factor analysis typically leads to the emergence of a number of factors that are used to facilitate a simplified explanation of the associations captured in the correlation matrix yielded from a data set [26]. Traditional factor analysis used the technique to cluster a group of variables (or scale items) into a factor as an alternative manifestation of these variables (or scale items). Therefore, the purpose of data reduction was obtained, and a simplified explanation of data was revealed by factor analysis. On the other hand, Q factor analyses generated clusters of persons, rather than clusters of Q statements, by using the PQ Method (V2.35) [27]. Each resulting final factor represented a group of individuals with similar perspectives. Further, participants were clustered into groups (factors) according to the similarities of their Q sorts [25]. In other words, a group of persons who share a similar perspective emerged. A specific composite Q sort, which hypothetically represented the group perspective, was derived for each of the final factors [28].

Finally, the Q methodology uses characteristic statements that are ranked at the extremes of a composite Q sort, which enables the interpretation of each identified group perspective (factor) [17]. Due to the strengths of grouping participants according to their perspective tendency [27], the compared perspectives of the supervisory interaction can be adequately addressed and illustrated.

#### 2.2.3. Q Statements

Designated Q statements were provided to address the raised issues related to drug use prevention during supervisory interactions. The statement sentences were developed according to a literature review [6,29,30,31,32,33,34,35,36,37,38,39] and experiences of youth counsellors. The research team discussed the draft statements with two senior youth counsellors; both worked with students of drug use problem for more than 10 years. After several group discussions, the statements were categorized as follows: strengthen information with personal relevance [6,36,37,38], health consequences of drug use [30,32,33], overcome barriers to change [6,28,33,34,35], development of preventive strategies [35,36,37,38], and supportive relationships [29,35,39]. The statements were later reviewed by three health professionals with expertise on drug use to ensure their appropriateness. The areas of academic expertise of the health professionals included health education, nursing, and public health. They were particularly experienced in conducting school-based drug use prevention programs and had published research articles on substance use prevention. Finally, a set of 39 Q statements was compiled (Table 1).

### 2.3. Data Collection

The study was conducted after receiving ethical approval. The research team visited the relevant schools to explain and demonstrate to participants how Q sorting was completed. Subsequently, supervisory staff and students met separately in quiet rooms. Only a single participant was allowed in each time slot. All participants completed a paper-based questionnaire to obtain background information before Q sorting.

**Figure 1:**
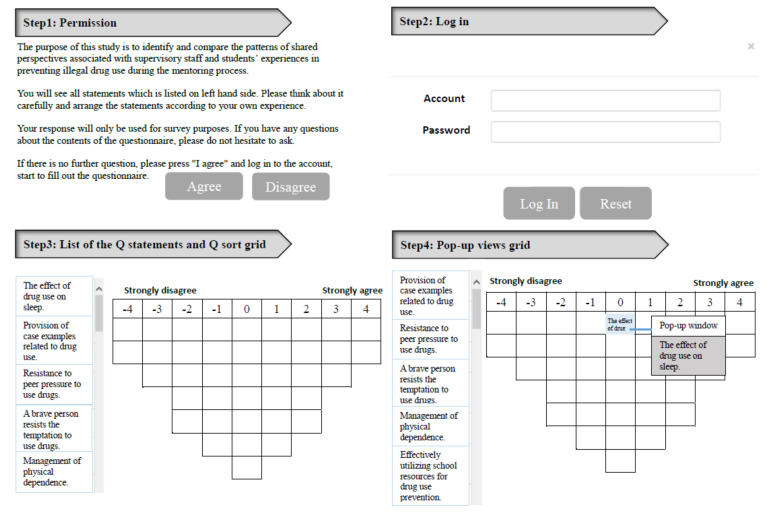
Illustration of online Q sort procedure. The content of this interface was translated into English; the original interface was in Chinese.

Figure 1 illustrates the user interface for the online Q sort procedure. Participants were provided with a laptop as well as account names and passwords to perform the sorting tasks. They were asked to fill their Q sort in a Q sort grid, generating a distribution of degrees from +4 (strongly agree) to −4 (strongly disagree) statements. The Q sorting helped to clarify the participants’ subjective opinions on these statements.

The Q sort task required the participants to consider the statements according to their experiences with drug use prevention. Participants were asked, “In order to prevent drug use, are the following statements important to you during the supervisory interactions?” We advised the participants to initially divide the Q statement into three groups (positive, negative, or neural). We provided a printed list of Q statements so that each statement could be marked as positive or negative. If a statement could not be classified as positive or negative, then it was classified as neutral. When complete, participants refined the positive-ranked statement group with degrees from +4 to +1 and assigned each statement to cells in the grid based on the number of degrees determined. Participants used the mouse to move each statement from the left panel to right panel in the Q sort grid until all positive rank statements were placed. Participants then repeated the process on the left side of the Q sort grid for the negative statements. If the positive and negative order cells were not filled, participants selected statements from the neutral group and placed them in empty cells. The remaining statements were placed in the middle column which was ranked “0”. Participants could adjust the position of statements repeatedly until they felt comfortable.

Participants (supervisory staff and students) received a coupon worth NTD$200 as an appreciation for their provision of Q sorts.

### 2.4. Statistical Analysis

PQ Method 2.35 was used to analyze the data collected from Q sorts [39]. The PQ Method software is a statistical program dedicated to the statistical analysis of Q studies (http://schmolck.org/qmethod/). Electronic data from the participants’ online Q sorts were imported to PQ Method software for analysis. Q factor analysis was applied to constitute supervisory staff and student groups based on the similarities in Q sorts. Further, a scree plot was employed to determine the number of retained factors. Finally, Q factor analyses were separately performed for supervisory staff and students.

### 2.5. Sample Size Estimation

In this study, sample size estimation was performed according to two rules [25]. First, there was one participant for every three Q statements. Second, it was suggested that at least three participants load highly on each perspective. According to the first rule, there were 39 Q statements, and at least 13 participants were expected for each group of supervisory staff and students. According to the second rule, the study revealed five perspectives, due to which at least 15 participants were included in each group of supervisory staff and students. However, it was impossible to know in advance the number of perspectives that would be the best solution for the Q factor analysis. Therefore, 31 participants in each group was considered sufficient because the suggested number was doubled (15 × 2 = 30) according to the aforementioned calculation.

## 3. Results

### 3.1. Characteristics of Study Participants

As shown in Table 2, supervisory staff included 23 men and eight women. Their mean age was 38.61 years (SD = 5.60). The duration of services as a supervisory staff and employment was 3.07 and 8.75 years, respectively. Similarly, the majority (85.71%) of the students were male students. Most (77.42%) were older than 18 years, with a mean age of 18.26 years (SD = 1.46). More than half (58.06%) of the students lived with both parents.

### 3.2. Comparison of Characterizing Statements between Groups

Q factor analyses were separately performed for supervisory staff and students. Following analysis, five-factor solutions with eigenvalues above 1 were extracted for both supervisory staff and students. The result of the five-factor solution for supervisory staff accounted for 58% of the total variance. The supervisory staff were clustered into five groups (five-factor solution) according to the similarities of their Q sorts. Another five-factor solution represented five groups of students with similar perspectives. This explained 49% of the total variance.

**Figure 2:**
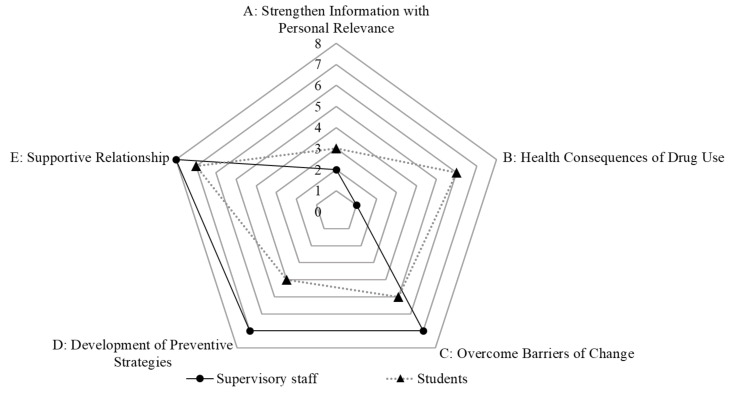
A glance at the similarities and differences between the perspectives of supervisory staff and supervisory staff by using a radar chart. The marks represent the number of characterizing statements for each category and indicated the degree of categories which were endorsed by the supervisory staff and students.

Q factor analyses generated clusters of individuals with similar perspectives. Hence, we used the term “groups” to indicate the factors resulting from Q factor analyses. Composite Q sorts of the resulting factors were used to interpret identified group perspectives. The characterizing statements, ranked at the most positive ends of each composite Q sort (+3 and +4), were used to illustrate perspective patterns of the participants who significantly loaded onto the specific group. Within each group, there were five characterizing statements, including three +3 and two +4 statements. A total of 25 characterizing statements were revealed for each group of supervisory staff and students (Table 3 andTable 4). We applied a radar chart to obtain an overview of the similarities and differences between supervisory staff and student groups (Figure 2). The marks in the figure represent the total numbers of characterizing statements for each category and indicate the degree to which the categories were endorsed by the supervisory staff and students. The farther the mark from the center, the more evident the perspectives associated with the respective category.

According to Figure 2, the most evident perspective was associated with “Supportive Relationship,” where eight and seven characterizing statements were located for supervisory staff and students, respectively. The least evident perspective was associated with “Strengthen Information with Personal Relevance,” where two and three characterizing statements were located for the supervisory staff and students, respectively. The different perspectives between supervisory staff and students were associated with “Health Consequences of Drug Use,” where only one characterizing statement was located for supervisory staff, but six characterizing statements for students.

### 3.3. Results of Q Factor Analysis: Supervisory Staff

The Q factor analysis revealed five groups of supervisory staff perspectives. These perspectives are discussed in the following subsections.

#### 3.3.1. Group 1: Importance of Friends’ Influences

Supervisory staff group 1’s perspectives included “Enabling students to become aware of the influences of friends with drug use problems” (+4), “Not hanging out with friends who have drug use problems” (+3), “Avoiding situations that may increase risks of using drugs” (+3), “Strategies to make friends who are positive influences” (+4), and “Enhancing the involvement of significant others to help students quit drugs” (+3). Five participants significantly loaded on this group.

#### 3.3.2. Group 2: Importance of Guidance Provision

Supervisory staff group 2 agreed with the importance of “Working with students to effectively manage the underlying reasons for their drug use” (+3), “Strategies to make friends with positive influences” (+3), and “Enhancing the involvement of significant others to help students quit drugs” (+4). Further, they agreed with the importance of “Provision of examples related to drug use” (+3) and “Enabling students to become aware of the influences of friends with drug use problems” (+4). Nine participants significantly loaded on this group.

#### 3.3.3. Group 3: Importance of Strategy Development

Supervisory staff group 3 agreed with the importance of “Strategies to strengthen willpower to resist temptation to use drugs” (+3), “Management for preventing relapse, especially strategies for coping with low mood” (+3), and “Avoiding situations that may increase chances of using drugs” (+3). Further, they agreed with the “Provision of examples related to drug use” (+4) and “Working with students to effectively manage the underlying reasons for their drug use” (+4). Six participants significantly loaded on this group.

#### 3.3.4. Group 4: Importance of Overcoming Barriers

Unlike group 3, supervisory staff group 4 expressed perspectives to overcome barriers of change. The characterizing statements were “Enabling students to become aware of the influences of friends with drug use problems” (+4), “Resisting a boy/girlfriend’s influences to use drugs” (+3), and “Not hanging out with friends who have drug use problems” (+3). They agreed with the importance of “The effect of drug use on behaviors” (+3) and “Enhancing the involvement of significant others to help students quit drugs” (+4), as well. Three participants significantly loaded on this group.

#### 3.3.5. Group 5: Importance of Management skills

The characterizing statements of supervisory staff group 5 included “Management for preventing relapse, particularly strategies for coping with low mood” (+3), “Management of physical dependence” (+4), and “Management of mental dependence” (+4). They agreed with “Enabling students to become aware of the influences of friends with drug use problems” (+3) and “Working with students to effectively manage the underlying reasons for their drug use” (+4), as well. Three participants significantly loaded on this group.

### 3.4. Results of Q Sort Analysis: Students

Among students, five groups of perspectives emerged from the Q factor analysis. These perspectives are discussed in detail in the following subsections.

#### 3.4.1. Group 1: Importance of Behavior Consequences and Assistances

Student group 1 emphasized statements such as “Provision of examples related to drug use” (+3), “A brave person resists the temptation to use drugs” (3), and “The effect of drug use on daily activities” (+4). Student group 1 agreed with “Educational needs of assisting students to find purpose in life” (+4) and “Effectively utilizing school resources for drug use prevention” (+3), as well. Seven participants significantly loaded on this group.

#### 3.4.2. Group 2: Importance of Interpersonal Influences

The students associated with Group 2 agreed with the importance of “Not hanging out with friends who have drug use problems” (+3), “Avoiding situations that may increase chances of using drugs” (+3), “Educational needs of leisure skill building and developing ability to engage in appropriate activities” (+3), “Strategies to make friends who are positive influences” (+4), and “Enhancing the involvement of significant others to help students quit drugs” (+4). Nine participants significantly loaded on this group.

#### 3.4.3. Group 3: Importance of Health Consequences of Drug Use

Student group 3 emphasized the health consequences of drug use. The characterizing statements included “The effect of drug use on sleep” (+3), “… on mental health” (+3), “… on daily activities” (+3), and “… on behaviors” (+4). They agreed with the statement “Strategies to convince my friends to participate in drug rehabilitation program with me” (+4), as well. Five participants significantly loaded on this group.

#### 3.4.4. Group 4: Importance of Resistance to Negative Influences

Student group 4 emphasized statements such as “Resistance to the immediate benefits of drug use, such as excitement, pleasure, euphoria” (+4); “Resistance to the peer pressure to use drugs” (+3); and “Not hanging out with friends who have drug use problems” (+3). They agreed on “Encouraging self-determination to quit drugs forever” (+4) and “The effect of drug use on mental health” (+3), as well. Four participants significantly loaded on this group.

#### 3.4.5. Group 5: Importance of Supportive Relationship

Student group 5 agreed with the importance of “Resistance to the dependency on drug use” (+3), “Educational needs of assisting students to find purpose in life and enabling students to show potential” (+3), “Development of school bonding by enhancing regular school attendance” (+3), “Strategies to interact with the person who knows my history of drug use” (+4) and “Enhancing the involvement of significant others to help students quit drugs” (+4). Three participants significantly loaded on this group.

## 4. Discussion

### 4.1. Principal Results

The results of the analyses showed agreement on the importance of supportive relationships, for example, enhancing the involvement of significant others to help an individual quit drugs, for both supervisory staff and student groups. Earlier studies have revealed that the perceived lack of support from family and friends is a pivotal factor in adolescents’ risk of relapse [40]. One difference between the supervisory staff and student groups was that whereas students highlighted the importance of health consequences of drug use, supervisory staff did not. Introducing the health consequences of drug use frequently has been applied as a motivation strategy for behavior change [41]. An enhanced perception of the negative outcomes of drug use can elicit discrepancies between individuals’ behaviors and goals and motivate them to make behavioral changes [10,42]. Unlike students, supervisory staff may feel that health hazards were knowledge-based information and had less impact on behavior change when compared to fostering positive attitudes or refusal skills. This difference between supervisory staff and students warrants further investigation.

Another interesting difference was that three Q statements were endorsed by more than half of the supervisory staff groups, whereas none were endorsed by more than half of the student groups. A possible reason is that supervisory staff training/supervising experience generated some degree of consensus across various supervisory staff groups. The staff emphasized the importance of interpersonal influence during interaction, as well. They strongly agreed that an alliance between supervisory staff and students could create a climate of trust and establish a necessary supportive relationship [43]. Supervisory staff firmly believe the key elements of provision of companionship, support, and guidance [14], regardless of the groups that they loaded onto.

Our findings revealed variabilities in supervisory practices. An earlier study revealed that adult perceptions influenced their practices of mentoring [44]. The perspectives of some staff, who loaded onto groups 1 and 4, were in line with earlier research in that peer influences were one of the most significant contributors to drug use among adolescents [6,45,46]. These staff emphasized external influences during supervision, whereas others prioritized internal influence. The staff loading onto groups 3 and 5 recognized the importance of assisting students to develop preventive strategies during supervision, such as coping with low mood [35,36,37] and management of physiological and psychological dependence [30,32,33].

Students who joined a supervisory program were expected to stay drug free; however, not all could fulfill this expectation [47]. The students associated with groups 1 and 3 emphasized the health consequences of drug use. In addition, self-encouragement to avoid the temptation to use drugs was emphasized. Examples could offer students opportunities to explore the pros and cons of behavioral consequences [8,48].

Students loading onto groups 2, 4, and 5 emphasized involving significant others, people who know the students’ history of drug use, and encouraging regular school attendance. Earlier studies have suggested that avoiding classes is significantly associated with drug use among vocational and senior high school students [45]. Our findings strengthen the importance of not only peer pressure avoidance but also encouragement of school attendance during supervision to prevent drug use. Students emphasized the development of an ability to engage in appropriate activities and a sense of purpose in life, as well. An individual’s purpose in life was proposed as a spiritual mechanism that contributed to his or her recovery from substance abuse and dependence [49].

### 4.2. Limitations

The current study has some limitations. In this study, strategies for assuring accuracy of Q sorting were that participants were asked to approve their final Q sort and were allowed to adjust the order of each statement if they felt it necessary. However, participants expressed that qualitative information associated with their Q sort would enhance appropriate interpretation of the study findings. A lack of qualitative data may decrease the degree of revealing in-depth participant thoughts. Further study is encouraged to include collection of qualitative information during the Q sort process. In addition, providing a comparison of gender differences would advance the understanding of the influence of youth-adult interaction. However, there were only three female students in the current study. Gender comparison is encouraged in studies with sufficient female sample size. Further, our study considered only in-campus, rather in-community, participants. All the participants were students of vocational or senior high schools. The adolescents who drop out of school may have different perspectives regarding drug use prevention than those attending schools. Hence, we suggest that future studies conduct a Q methodological survey among a sample of adolescents that includes those within the community.

## 5. Conclusions

The novel approach of using the Q sort methodology in the study helped uncover significant similarities and differences between the perceptions of supervisory staff and students. Regarding youth-adult interaction for drug use prevention, students highlighted the importance of the health consequences of drug use, whereas supervisory staff did not. Both students and supervisory staff emphasized that enhancing the involvement of significant others helps students quit drugs. In addition to involving significant others, supervisory staff emphasized the importance of recognizing peer influences during supervision. Further, the students pointed out that interactions with people who knew their history of drug use were important during supervision. Finally, supervisory staff emphasized the development of preventive strategies, whereas students emphasized enhancing their regular school attendance.

## 6. Implications for Practice

An examination of the similarities and differences between supervisory staff and students’ perceptions of supervisory interaction clarified how to support students in preventing drug use. While preventing students from drug use, enhancing the involvement of significant others is suggested as an approach to help students quit drugs. To gain insight into the effectiveness of youth-adult interactions, the study’s findings suggested a need to extend the dyadic relationships to include the students’ social contexts [44].

Supervisory staff practices vary according to whether they emphasize internal or external influences. Not all students who joined the supervisory program were ready to change themselves. In addition to fostering positive attitudes or refusal skills, a careful assessment of students’ needs is important for appropriately advising and supervising students for drug use prevention. Students who were not ready to change themselves required information on the health consequences of drug use [42], for example, statements advocating the value of change may motivate students to change their behaviors [50].

Some students emphasized the importance of promoting regular school attendance and interacting with people who know their history of drug use. However, supervisory staff did not recognize the importance of these two statements. Accordingly, counseling training programs or workshops might find the study’s findings helpful and emphasize the significance of conducting priority assessments of students. In addition to assessing students’ needs, understanding their priorities contributed to the establishment of effective adult-youth relationships in drug use prevention.

## Figures and Tables

**Table 1:** List of the Q statements.

The Categories and Subcategories of Q Statements Derived from Published Literature	Q Statements Derived from Group Discussion with Senior Counsellors and Reviewed by Academic Expertise
Category A: Strengthen Information with Personal Relevance [6,36,37,38]	
⮚ Message provision	Provision of examples related to drug use.
	Provision of adverse consequences of drug use to increase perceived threat.
	Provision of legal consequences related to drug use.
	Provision of available medical referral services related to drug use.
⮚ Value advocacy	The sooner you change, the more helpful it is to yourself.
	A brave person resists the temptation to use drugs.
	Encouraging self-determination to quit drugs forever.
	Emphasizing the importance of drug-free and zero tolerance policy on campus.
Category B: Health Consequences of Drug Use [30,32,33]	
⮚ Physical health effects	The effect of drug use on physical health.
	The effect of drug use on sleep.
⮚ Psychological health effects	The effect of drug use on personality.
	The effect of drug use on mental health.
⮚ Behavior effects	The effect of drug use on daily activities.
	The effect of drug use on working.
	The effect of drug use on behaviors.
Category C: Overcome Barriers of Change [6,28,33,34,35]	
⮚ Drug dependence	Resistance to the immediate benefits of drug use, such as excitement, pleasure, euphoria.
	Resistance to the dependency on drug use.
⮚ Peer influences	Enabling students to become aware of the influences of friends with drug use problem.
	Resistance to peer pressure to use drugs.
	Resistance to the boy/girlfriend’s influences to use drugs.
	Not hanging out with friends who have drug use problems.
	Avoiding visiting friends who have drug use problems.
⮚ sensation seeking	Resistance to use drugs by increasing impulse control.
	Resistance to use drugs by thinking thoughtfully while encountering friends’ provocations to use.
Category D: Development of Preventive Strategies [35,36,37,38]	
⮚ Management skills for preventing drug use	Strategies to strengthen willpower to resist temptation to use drugs.
	Management for preventing relapse, especially strategies for coping with low mood.
	Management of physical dependence.
	Management of mental dependence.
	Avoiding situations that may increase risks of using drugs.
⮚ Educational needs	Educational needs of leisure skill building and developing ability to engage in appropriate activities.
	Educational needs for students to manage their allowance.
	Educational needs of assisting students to find purpose in life.
Category E: Supportive Relationship [29,35,39]	
⮚ School bonding	Working with students to effectively manage the underlying reasons for their drug use.
	Development of school bonding by enhancing regular school attendance.
	Effectively utilizing school resources for drug use prevention.
⮚ Positive supports	Strategies to interact with the person who knows student’s history of using drugs.
	Strategies to convince student’s friends to participate in drug rehabilitation program with me.
	Strategies to make friends with positive influences.
	Enhancing involvement of significant others to help students quit drugs.

**Table 2:** Background information of the supervisory staff and students.

Supervisory Staff (*n* = 31)	
Gender, *n* (%)	
Male	23 (74.19%)
Female	8 (25.81%)
Age	38.61 ± 5.60
Duration of service as a supervisory staff (years)	3.07 ± 1.61
Duration of employment (years)	8.75 ± 7.28
**Students (*n* = 31)**	
Gender, *n* (%)	
Male	28 (85.71%)
Female	3 (14.29%)
Age	18.26 ± 1.46
Age, *n* (%)	
< 18 years old	7 (22.58%)
≥ 18 years old	24 (77.42%)
Status of living with parents, *n* (%)	
Live with both parents	18 (58.06%)
Live with single parents	10 (32.26%)
Not living with any parent	3 (9.68%)

**Table 3:** Characterizing statements—supervisory staff.

Categories of Q Statements	Group 1 Importance of Friends’ Influences	Group 2 Importance of Guidance Provision	Group 3 Importance of Strategy Development	Group 4 Importance of Overcoming Barriers	Group 5 Importance of Management skills
A: Strengthen information with personal relevance (two statements ^a^)		Provision of examples related to drug use. (3)	Provision of examples related to drug use. (4)		
B: Health consequences of drug use (one statement ^a^)				The effect of drug use on behaviors. (3)	
C: Overcome the barriers of change (seven statements ^a^)	Enabling students to become aware of the influences of friends with drug use problem. (4) Not hanging out with friends who have drug use problems. (3)	Enabling students to become aware of the influences of friends with drug use problems. (4)		Enabling students to become aware of the influences of friends with drug use problems. (4) Resistance to the boy/girlfriend’s influences to use drugs. (3) Not hanging out with friends who have drug use problems. (3)	Enabling students to become aware of the influences of friends with drug use problems. (3)
D: Development of preventive strategies (seven statements ^a^)	Avoiding situations that may increase risks of using drugs. (3)		Strategies to strengthen willpower to resist temptation to use drugs. (3) Management for preventing relapse, especially strategies for coping with low mood. (3) Avoiding situations that may increase risks of using drugs. (3)		Management for preventing relapse, especially strategies for coping with low mood. (3) Management of physical dependence. (4) Management of mental dependence. (4)
E: Supportive relationship (eight statements ^a^)	Strategies to make friends with positive influences. (4) Enhancing involvement of significant others to help students quit drugs (3).	Working with students to effectively manage the underlying reasons for their drug use. (3) Strategies to make friends with positive influences. (3) Enhancing involvement of significant others to help students quit drugs. (4)	Working with students to effectively manage the underlying reasons for their drug use. (4)	Enhancing involvement of significant others to help students quit drugs. (4)	Working with students to effectively manage the underlying reasons for their drug use. (3)

Note: ^a^ Total numbers of statements in each category. The numbers (4) and (3) in parentheses represent that the statements most accurately reflected the experience of participants who loaded significantly onto the given group.

**Table 4:** Characterizing statements—students.

Categories of Q Statements	Group 1 Importance of Behavior Consequences and Assistances	Group 2 Importance of Interpersonal Influences	Group 3 Importance of Health Consequences of Drug Use	Group 4 Importance of Resistance to Negative Influences	Group 5 Importance of Supportive Relationship
A: Strengthen information with personal relevance (three statements ^a^)	Provision of examples related to drug use. (3) A brave person resists the temptation to use drugs. (3)			Encouraging self-determination to quit drugs forever. (4)	
B: Health consequences of drug use (six statements ^a^)	The effect of drug use on daily activities. (4)		The effect of drug use on sleep. (3) The effect of drug use on mental health. (3) The effect of drug use on daily activities. (3) The effect of drug use on behaviors. (4)	The effect of drug use on mental health. (3)	
C: Overcome the barriers of change (five statements ^a^)		Not hanging out with friends who have drug use problems. (3)		Resistance to the immediate benefits of drug use, such as excitement, pleasure, euphoria. (4) Resistance to the peer pressure to use drugs. (3) Not hanging out with friends who have drug use problems. (3)	Resistance to the dependency on drug use. (3)
D: Development of preventive strategies (four statements ^a^)	Educational needs of assisting students to find purpose in life. (4)	Avoiding situations that may increase risks of using drugs. (3) Educational needs of leisure skill building and developing ability to engage in appropriate activities. (3)			Educational needs of assisting students to find purpose in life and enabling students to show potential. (3)
E: Supportive relationship (seven statements ^a^)	Effectively utilizing school resources for drug use prevention. (3)	Strategies to make friends with positive influences. (4) Enhancing involvement of significant others to help students quit drugs. (4)	Strategies to convince my friends to participate in drug rehabilitation program with me. (4)		Development of school bonding by enhancing regular school attendance. (3) Strategies to interact with the person who knows my history of using drug. (4) Enhancing involvement of significant others to help students quit drugs. (4)

Note: ^a^ Total numbers of statements in each category. The numbers (4) and (3) in parentheses represent that the statements most accurately reflected the experience of participants who loaded significantly onto the given group.
